# Biocompatibility analysis and chemical characterization of Mn-doped hydroxyapatite

**DOI:** 10.1007/s10856-023-06744-0

**Published:** 2023-07-29

**Authors:** L. S. Villaseñor-Cerón, D. Mendoza-Anaya, S. López-Ortiz, R. Rosales-Ibañez, J. J. Rodríguez-Martínez, M. I. Reyes-Valderrama, V. Rodríguez-Lugo

**Affiliations:** 1 Área Académica de Ciencias de la Tierra y Materiales, Instituto de Ciencias Básicas e Ingeniería, Universidad Autónoma del Estado de Hidalgo, Carretera Pachuca-Tulancingo Km. 4.5, 42184 Pachuca, Mexico; 2 Instituto Nacional de Investigaciones Nucleares; Carr. México-Toluca s/n La Marquesa, C.P. 52750 Ocoyoacac, Estado de México México; 3 Instituto de Física, Universidad Autónoma de San Luis Potosí, Av. Parque Chapultepec1570, Privadas del Pedregal, San Luis Potosí, SLP México; 4 Laboratorio de Ingeniería Tisular y Medicina Traslacional, Facultad de Estudios Superiores Iztacala, Universidad Nacional Autónoma de México. Avenida Tenayuca-Chalmita S/N, Cuautepec Barrio Bajo, Alcaldía Gustavo A. Madero, CP. 07239 Ciudad de México, México

## Abstract

The present work studies the effect of Mn doping on the crystalline structure of the Hap synthesized by the hydrothermal method at 200 °C for 24 h, from Ca(OH)_2_ and (NH_4_)_2_HPO_4_, incorporating MnCl_2_ to 0.1, 0.5, 1.0, 1.5 and 2.0 %wt of Mn concentrations. Samples were characterized by the X-Ray Diffraction technique, which revealed the diffraction peaks that corresponded to the hexagonal and monoclinic phase of the Hap; it was observed that the average size of crystallite decreased from 23.67 to 22.69 nm as the concentration of Mn increased. TEM shows that in all samples, there are two distributions of particle sizes; one corresponds to nanorods with several tens of nanometers in length, and the other in which the diameter and length are very close. FTIR analysis revealed absorption bands corresponding to the PO_4_^−3^ and OH^−^ groups characteristic of the Hap. It was possible to establish a substitution mechanism between the Mn and the ions of Ca^+2^ of the Hap. From the Alamar blue test, a cell viability of 86.88% ± 5 corresponding to the sample of Hap at 1.5 %wt Mn was obtained, considered non-cytotoxic according to ISO 10993-5. It also evaluated and demonstrated the good osteoinductive properties of the materials, which were verified by histology and immunofluorescence expression of osteogenic markers. Adhesion, viability, biocompatibility and osteoinductive properties, make these materials candidates for future applications in bone tissue engineering with likely uses in regenerative medicine.

**Graphical Abstract:**

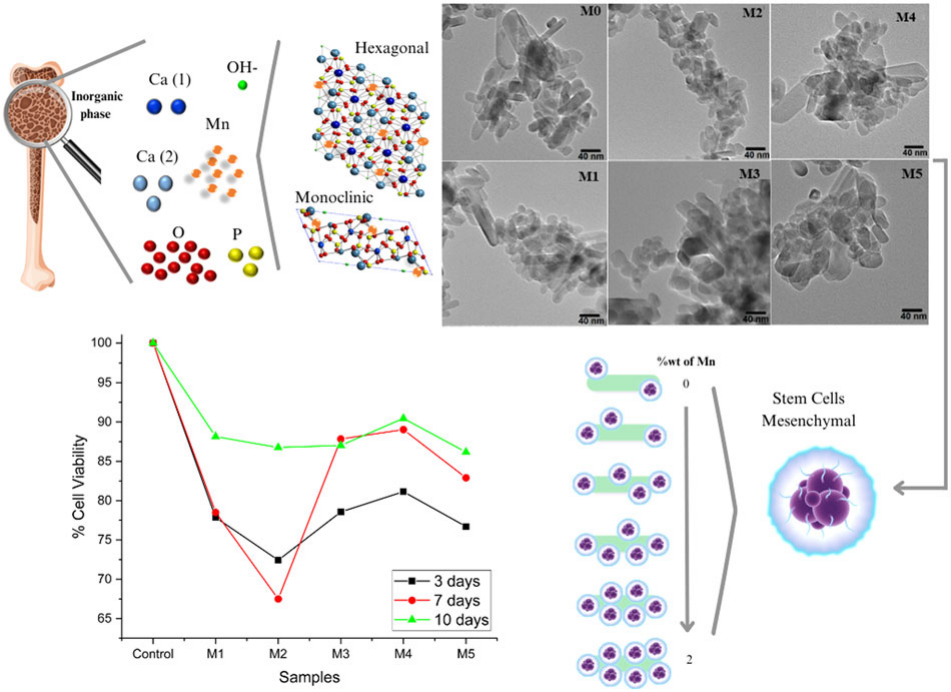

## Introduction

Osseointegration is an essential property within biomaterials since it consists of a biological process that causes the implant to integrate and unite with the bone tissue, where different factors intervene, such as a) biocompatibility, b) design, c) type of surface, d) host state, e) the surgical insertion technique and f) those applied to the implant [1, 2]. In orthopedic and dental implants, a coating is required that facilitates osseointegration and prevents a bacterial infection (peri-implantitis), one of the most advanced forms of loss of integration with bone in the tissue. According to estimates, this infection impacts 10% of implants over five years old [3]. In tissue engineering and regenerative medicine, the considerable loss of bone tissue represents a challenge for modern medicine. When this happens, and the body cannot repair it, different strategies have been used to replace this irreversible loss. Bone tissue allografts and autografts are the gold standard for treating bone lesions. However, they have risks and limitations, such as the amount of tissue available, immune reactions, morbidity at the donor site, and risks of disease transmission [4]. Various synthetic biomaterials are used in regenerative medicine, such as bioceramics and biopolymers [1]. In particular, bioceramics are a unique set of fully, partially, or non-crystalline ceramics that are non-metallic inorganic materials [5, 6], designed for repairing and reconstructing diseased body parts and have high potential as healing scaffolding. Traditionally, bioceramics have been used to fill and restore bone defects since they have desirable biocompatibility characteristics, bioactivity, mechanical properties, and resistance to heat and corrosion [6, 7]. In addition, ceramics are available in a wide variety and large quantities. In tissue engineering, scaffolds based on these materials are being developed to regenerate hard tissues, and their effects on soft tissues are even being investigated [8, 9]. Chiroff et al. first recognized that corals made by marine invertebrates have a structure similar to that of cortical and cancellous bone and that they might be suitable grafts for skeletal application [10]. Since then, different ceramics have been used in bone regeneration. Some bioceramics, such as hydroxyapatite (Hap, Ca_10_(PO_4_)_6_(OH)_2_) and alumina, are intended to be permanent devices; thus, they do not release their components and are expected not to generate toxic reactions in the human body, [6, 11]. Specifically, Hap is a bioceramic of great interest within the medical field due to its excellent properties, such as osseointegration, biocompatibility, and bioactivity, among others, which facilitates its application in the transport of drugs and the reconstruction of damaged tissue from grafts or implants [12–14].

Scientific reports show that Hap induces a bioactive surface of implants, increasing up to 70% of the amount of bone that comes into contact with the implant [15–21]. Therefore, it is used as a coating for orthopedic implants, where a strong interaction of the bone with the current environment is required. It has also been reported that nanocrystalline Hap has better bioactivity, biocompatibility, and improved mechanical properties than microcrystalline Hap. Then, the morphological control of synthetic Hap is another aspect of great interest because it can improve cell binding and mineralization in vivo, suggesting that Hap, with morphological and dimensional control, is a better candidate for developing biomaterials [22, 23].

Today several methods have been developed to synthesize hydroxyapatite for its application as bioceramic. Each requires several experimental parameters, such as pH, temperature, and the molar ratio of chemicals, to produce the Hap with specific characteristics: chemical purity, morphology, particle size, crystallinity, porosity, etc. Methods of synthesis include the following: dry methods (solid state and mechanosynthesis) [21, 24–27], high-temperature processes (pyrolysis and others) [28–38], wet (sol-gel, hydrothermal and others) [34, 37, 39]. In particular, the hydrothermal method synthesizes at temperatures and pressures above 1 atmosphere and 100 °C to obtain regular, homogeneous, and crystalline nanostructures [40–44].

Although pure Hap can be applied as bioceramic material, it seeks to improve its properties, such as mechanical, biocompatibility, bioactivity, and osseointegration, to enlarge their applications for bone replacement or implant coatings. To optimize these properties, bivalent and trivalent minerals can be incorporated into the crystal structure during its synthesis, in which substitution by Ca^+2^ ions can be carried out; ions of Sr^+2^, Mg^+2^, Mn^+2^, Cd^+2^, Na^+^, and others and others can replace these. In the same way, for PO_4_^−3^ groups are susceptible to being replaced by anions such as CO_3_^−2^, SO_3_^−2^, etc. OH^-^ ions can be replaced by F^−^ and Cl^−^ ions. That means doping the Hap allows for modifying its crystalline structure, improving its structural stability and biocompatibility, osteoconduction, osseointegration, antibacterial, and luminescence [39, 45–52].

It has been reported that Mn influences the morphology, particle size, and lattice parameters, improving the mechanical properties and densification of the Hap without altering its stability [46, 53–55]. Kandori et al. observed that adding 0.20 mol Mn during the synthesis decreases the size of the rod structures of pure Hap in diameter and length from 23 and 55 nm to 10 and 49 nm, respectively [56]. Pandya et al. synthesized Mn-doped Hap with a nano rod-like in structure, observing that the dopant influences the structural and morphological behavior of Mn-doped Hap; even more, they reported that the crystallite size increases with an increase in the concentration of dopant [55]. Nawawi et al. analyzed the behavior of biphasic calcium phosphate phases at different concentrations of Mn (0, 0.01, 2, 5, and 15 %moles), noting a change in the degree of crystallinity of the tricalcium phosphate phase, being more intense at a concentration of 5 %mol [57]. On the other side, Robles-Águila synthetized Hap powders doped with metal ions (M+ = Mn^2+^, Fe^3+^, Ni^2+^), through co-precipitation and hydrothermal-assisted sol-gel method; their XRD results indicated the presence of the hexagonal Hap phase in pure and doped samples, showing that doping does not affect the volume of the hexagonal unit cell [15]. On the other hand, Mayer et al. prepared Mn-doped Ca-deficient Hap samples by precipitation method; although these Hap samples present the characteristic hexagonal apatite structure, the morphology depends on the Mn concentration; if the Mn content is 0.73%, platelet crystals about micron size and needle-like crystals up to 100 nm were observed; while with a content of 1.23%, crystals were smaller, needle-like and with sizes up to 400 nm only [58]. In a recent scientific paper, Huan Liu et al. showed that incorporating Mn into the structure of Hap synthesized by the co-precipitation method leads to a linear decrease in particle size with increasing Mn content; in addition, the interplanar spacing of (002) also decreases linearly [59].

Incorporating Mn ions into the crystal structure of Hap allows directing the material to a medical application field due to Mn’s properties: increased biocompatibility and improved cell propagation and viability. In addition, Mn influences the adhesion between bone cells and implant material, which favors its application in bone implants.

The present work focuses on studying the effect of the incorporation of Mn in the crystal structure of the Hap synthesized by the hydrothermal method. Validated assays using Alamar blue to evaluate its cell viability, osteogenic differentiation was performed, and the expression of osteogenic markers by immunofluorescence to determine the biocompatibility and cell adhesion of matter were also made.

## Materials and methods

### Materials

Ammonium phosphate dibasic [(NH4)2HPO4, 98.5%] brand Meyer and calcium hydroxide [Ca(OH)2, 98%)] brand Sigma Aldrich were used as precursors for the synthesis of Hap. In contrast, the initial synthesis process added nitric acid (HNO3, 2 M) brand Meyer to the pH control. Manganese chloride (MnCl2) was used to synthesize HAp: Mn as a dopant.

### Methods

#### Synthesis of pure and doped Hap

**Fig. 1:**
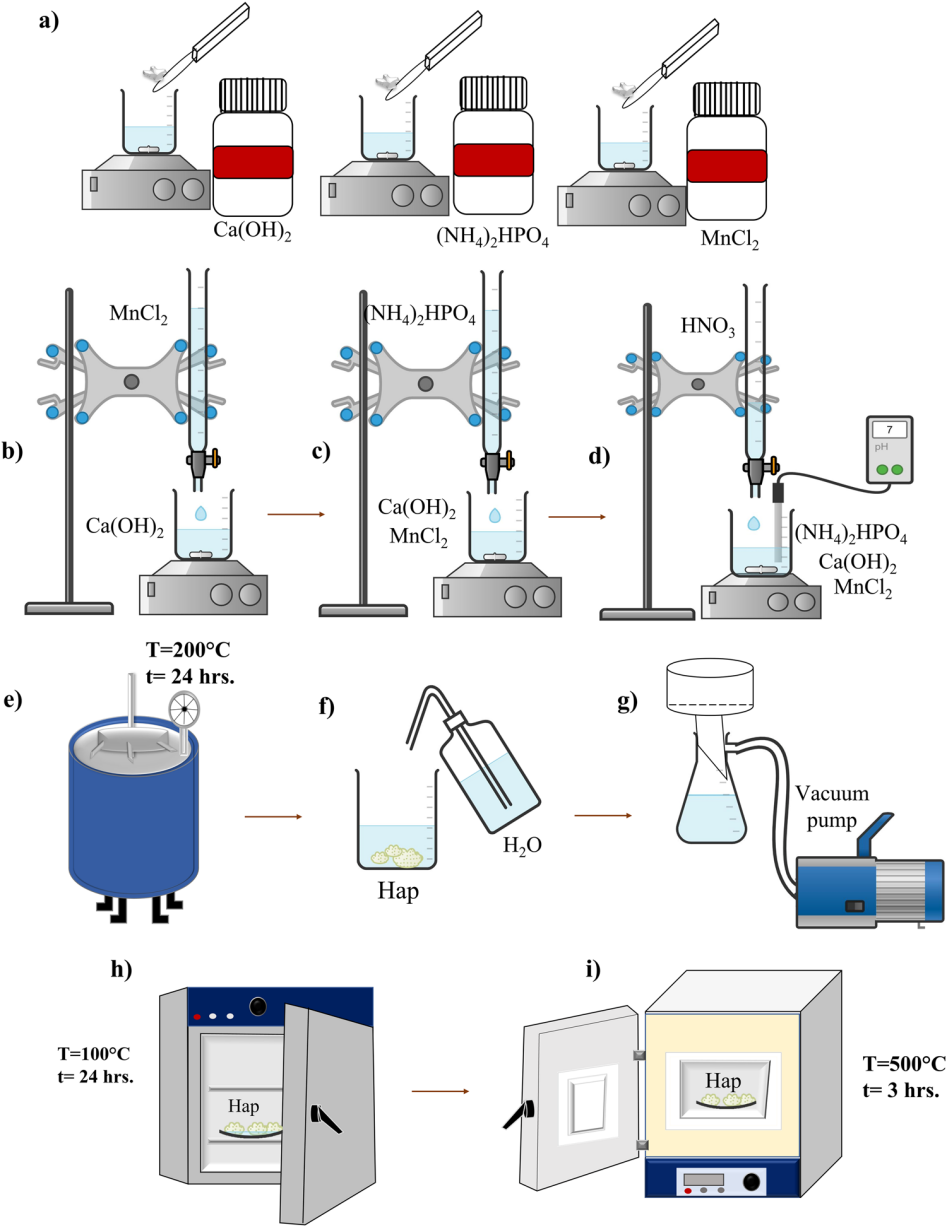
Experimental design for the synthesis of modified hydroxyapatite with different percentages of MnCl_2_. **a** Dissolution of precursors, **b** incorporation of MnCl_2_ into the Ca solution, **c** aggregation of the PO_4_^−3^ precursor, **d** pH adjustment, **e** synthesis of the material, **f** washing of the sample, **g** overrun of the sample by means of an vacuum pump, **h** drying of the sample and **i** thermal treatment

Figure 1 presents the experimental design for the synthesis of pure and doped Hap from the dissolution of the precursors using the hydrothermal method: 8 g of Ca(OH)_2_ and 12.33 g of (NH_4_)_2_HPO_4_, each diluted separately in 30 ml of deionized water at a constant stirring of 250 rpm and finally the dissolution of the MnCl_2_ dopant at different concentrations (0, 0.1, 0.5, 1.0, 1.5 and 2.0 %wt). The samples were identified as: M0, M1, M2, M3, M4 and M5, respectively) in 10 ml of deionized water (Fig. 1a). Consecutively, the solution of MnCl_2_ was added by dripping into the Ca(OH)_2_ solution at a constant stirring of 250 rpm per 10 min (Fig. 1b), and (drop by drop) the (NH_4_)_2_HPO_4_ solution was gradually added to the Ca(OH)_2_/MnCl_2_ solution (Fig. 1c), by drip. Subsequently, 45 ml of HNO_3_ was added to modify the pH of the solution to 7 (Fig. 1d). Subsequently, the solution was placed inside an autoclave at a temperature of 200 °C for 24 hours (Fig. 1e). After that, a washing process of the synthesized samples was carried out with deionized water and filtered under vacuum (Fig. 1f, g). Then, samples were dried at 80 °C for 8 h (Fig. 1h) and calcined at 500 °C for 3 h (Fig. 1i).

#### Characterization

The samples were characterized by X-ray diffraction (XRD) techniques to identify the presence of crystalline phases in pure and doped Hap samples. A diffractometer of the Bruker brand Discover D8 model with a CuKα radiation target (l = 1.54051 Å) was used. XRD data were acquired from 10° to 80° in 2q degree, 40 KV, 40 mA, and 0.03° of step size. The XRD patterns were refined and analyzed by the multiphase Rietveld method using the TOPAS code to determine the amounts (% fraction) of the different phases. In this method, the least-squares refinements are carried out until the best fit between the experimental and theoretical XRD patterns is obtained, considering the presence of all previously identified crystalline phases.

For the morphological and elemental chemical characterization, the Transmission Electron Microscope (TEM), JEOL model 2010F with an electron energy beam of 200 KeV and equipped with a Noran energy-dispersive X-ray spectroscope (EDS) was used. Likewise, the Fourier transform infrared spectroscopy (FTIR) analysis was performed in a range of 4000 to 450 cm^−1^, with a step of 2 cm^−1^ using a spectrometer Perkin Elmer Ft-Ir System Spectrum Gx with NIR FT-Raman. The samples were prepared using a microgram of sample powder mixed with KBr to prepare semi-transparent tablets; the FTIR spectrum was graphed using the Origin software.

#### Cellular viability by Alamar blue assay

AlamarBlueTM Cell Viability, Invitrogen, USA was used to determine cell viability. The working protocol for ceramic powder is described below. Human dental pulp stem cells (hDPSCs) were seeded in a 96-well culture dish (Techno Plastic Products-TPP, Switzerland) at a density of 5 × 10^3^ in quintuples, then incubated for 48 hours at 37 °C and 5% CO_2_ to allow adhesion to the substrate in the culture box. After the incubation time, a solution of 1 mg/mL of Mn-doped Hap (0.1, 0.5, 1.0, 1.5, and 2.0 Mn wt%) in low glucose DMEM (Dulbecco’s Modified Eagle Medium Low-Glucose, Biow cest) was prepared; the hydroxyapatites were added to each one of the groups, and the cells only in the well served as a control group. Afterward, they were incubated at different times, 3, 7, and 10 days; once completed each time, the cells were incubated with a 100 µl solution of 10% Alamar blue for 4 hours at 37 °C protected from light. An Elisa plate reader was used to measure cell viability, equipped with a 570 nm and 630 nm filter.

#### Osteogenic differentiation

Approximately 5 × 10^3^ DPSCs per well in a 24-well plate (TPP) were seeded as follows: cells with low-glucose DMEM (Dulbecco’s Modified Eagle Medium Low-Glucose, Biowest) as a negative control group, cells with osteogenic medium (MesenCult™ Osteogenic Differentiation Kit (Human) # 05465) as a positive control group and experimental group cells with hydroxyapatite particles doped with different concentrations of 1.5% and 2% manganese (Mn). All groups were duplicated; the culture plate was placed in an incubator (Binder CB 150-UL) humidified atmosphere of 95% O_2_ and 5% CO_2_ at 37 °C. The osteogenic induction period was carried out for 21 days. Once the induction period was over, the culture medium was removed, and the cells were washed with distilled water. Fixation was carried out using 4% paraformaldehyde for 20 min at room temperature, and the fixative was removed and rewashed with distilled water to add the alizarin red stain (Alizarin Red S, Sigma # A5533) for 1 h at room temperature; finally, the stain is removed and immediately washed with distilled water. In addition, a phase contrast brightfield optical microscope (Leica DM IL LED) was used.

#### Osteogenic markers expression by immunofluorescence

To detect the expression of osteogenic markers RUNX-2 and collagen I, approximately 5 × 10^3^ DPSCs were seeded per well in a 24-well plate (TPP) in the same manner as above. Once the induction period was over, the culture medium was removed. The cells were washed with PBS 1X (Phosphate Buffered Saline, Biowest), later fixation was carried out using 4% paraformaldehyde for 20 min at room temperature, the fixative and rewashed with PBS, permeabilized with 0.05% triton X-100 for 30 min and blocked with 1% albumin for 45 min, washed with PBS and cells incubated overnight with RUNX2 monoclonal primary antibody (sc-360351, Santa Cruz) and antibody Collagen I monoclonal primary antibody (MA1-26771, Invitrogen) diluted 1:200 in albumin. The solution with the primary antibody was discarded. The FITC-anti-mouse secondary antibody (R&D Systems, USA) was added, diluted 1:200 in albumin, and incubated for 1 h at room temperature, protected from light. The secondary antibody was removed, and DAPI was added to stain nuclei 1:1000. Finally, for the acquisition of images, a Nikon A1R confocal microscope mounted on a stand of Nikon Eclipse Ti (Nikon Corporation, Tokyo, Japan) was also used.

## Results

### X-ray diffraction (XRD)

**Fig. 2:**
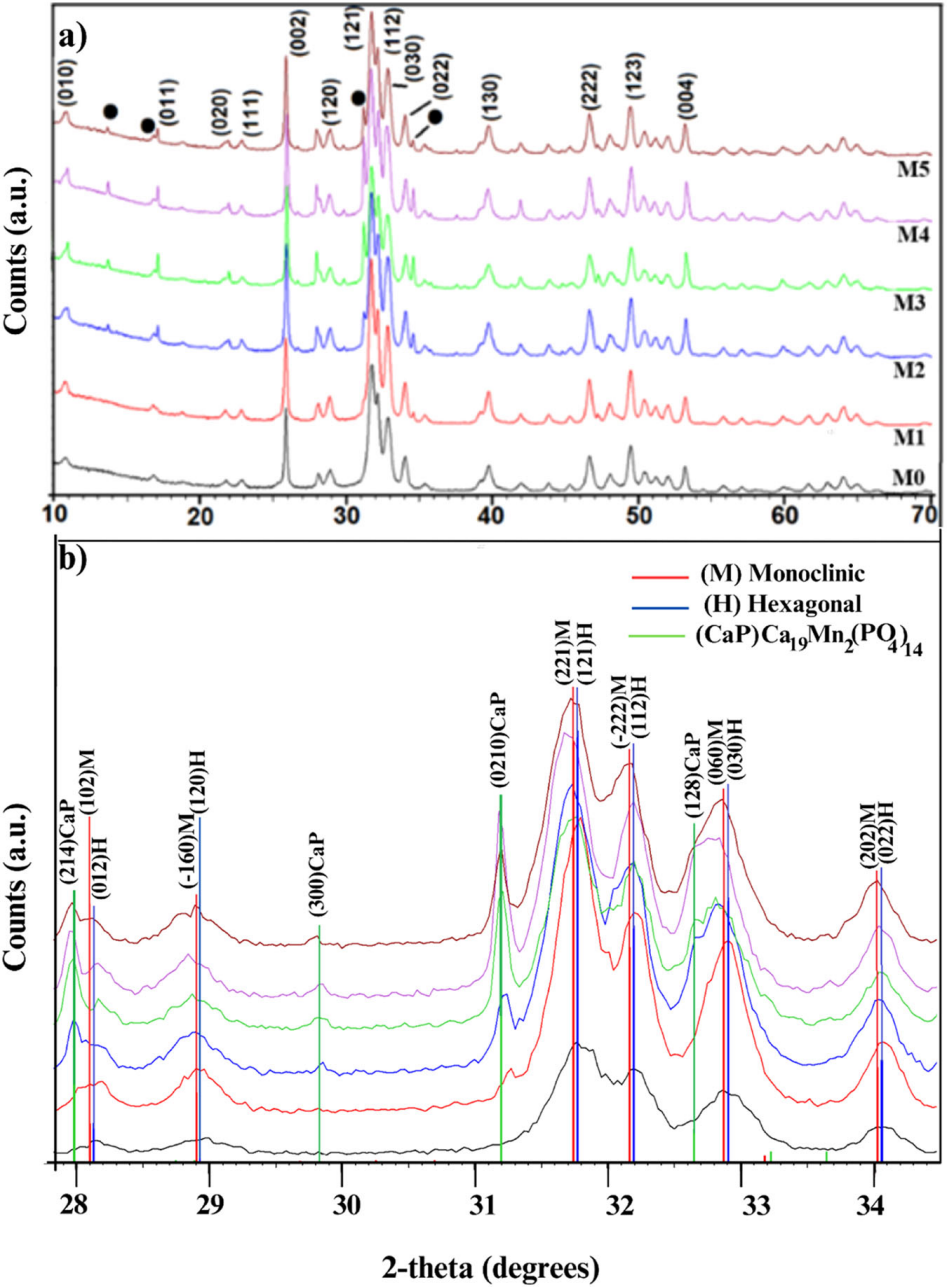
**a** XRD patterns from pure and Mn-doped hydroxyapatite (0, 0.1, 0.5, 1.0, 1.5, and 2.0 %wt of Mn). **b** Enlargement of the 2-theta range from 55.5° to 67°; it is possible to appreciate the contribution of each crystalline phase for two samples

Figure 2a shows the X-ray diffraction patterns corresponding to the Mn-doped Hap samples (M0, M1, M2, M3, M4, and M5). According to the ICDD (International Centre of Diffraction Data), the main diffraction peaks matched the hexagonal hydroxyapatite phase (Card No.09-432); the main *hkl* direction was pointed out for the corresponding X-ray diffraction peaks. Although XRD peaks overlap between hexagonal and monoclinic hydroxyapatite, a detailed analysis of the diffraction patterns showed the presence of the monoclinic Hap with an ICDD card No.76-0694 for all analyzed samples. This statement is supported in Fig. 2b, which is an enlargement of the 2-theta range from 28° to 34.5°, where it is possible to appreciate some diffraction peaks corresponding to the different crystalline phase identified; as can be seen in this figure, the diffraction peaks of monoclinic and hexagonal phases are very close. The formation of the Calcium manganese phosphate crystalline phase was also observed for Mn-doped Hap, as can be corroborated by the presence of the diffraction peaks at 13.70°, 29.84°, 31.19°, 34.59°, 41.25°, 44.80°, and 47.26° (Fig. 2), according to the card No. 00-048-1193; it was noted that the presence of this phase increases with increasing of Mn-dopant.

The X-ray diffraction results calculated the average crystallite size of the hydroxyapatite using the modified Sherrer equation proposed by A. Monshi et al. (Monshi et.al., 2012). This equation (Eq. (1)) relates to the crystallite size D_*hkl*_ with the X-ray incident wavelength (***λ***), the diffracted fullwidth at half-maximum (radians) caused by the crystallite size (***B***_***hkl***_ in radians) and the Bragg angle (***θ***); *hkl* are the Miller indices of the plane being analyzed.

1 $${{{\mathrm{D}}}}_{hkl} = {{{\mathrm{K}}}}\lambda /B_{hkl}\cos \theta _{hkl}$$

*K* is the crystallite-shape factor (a value of 0.89 is a good approximation). Applying the Scherrer equation (Eq. (1)) to the main diffraction peaks, a plot of ln**B**_***hkl***_ against ln(1/cos**θ**_***hkl***_) is obtained, and the intercept of a least squares line regression, ln*K***λ***/D* is obtained, from which a single value of D is determined.

**Fig. 3:**
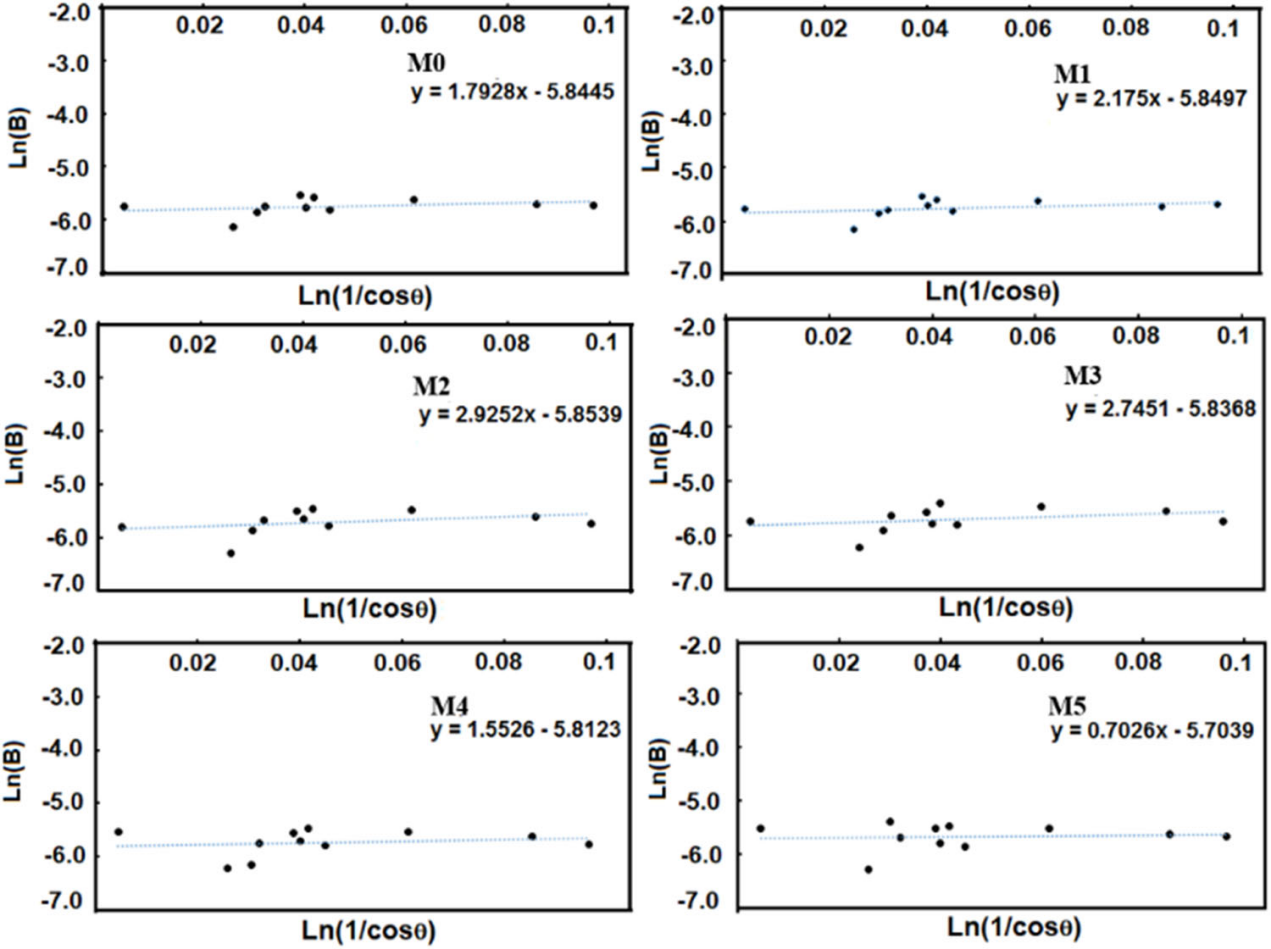
Linear plots of modified Scherrer equation and obtained intercepts for pure and doped Hap

In this work, the following *hkl* were used: (100), (111), (102), (210), (211), (112), (300), (202), (310), (222) and (213) to determine the crystallite size for each sample. Table 1 presents the corresponding values for pure Hap (M0), with which the plot of ln**B**_***hkl***_ against ln(1/cos**θ**_***hkl***_) was made. Similar tables were obtained for Mn-doped Hap samples, which were not included in the manuscript but were used to obtain the corresponding plots. Figure 3 indicates six plots of ln*β* against ln(1/cosθ) for individual pure and Mn-doped Hap, together with the linear least squares method equations obtained from linear regression using Excel software.

**Table 1:** Experimental values of 2θ, fullwidth at half-maximum (B in 2θ and radians), and calculated values of Ln(1/cos) and Ln(B) for pure Hap

hkl	2θ	B(2θ)	B(rad)	Ln(1/cosθ)	Ln(B)
(1 0 0)	10.809	0.358	0.006	0.005	−5.073
(1 1 1)	25.919	0.243	0.004	0.026	−5.460
(1 0 2)	28.17	0.32	0.006	0.031	−5.185
(2 1 0)	28.949	0.361	0.006	0.032	−5.064
(2 1 1)	31.801	0.449	0.008	0.039	−4.846
(1 1 2)	32.238	0.352	0.006	0.040	−5.090
(3 0 0)	32.922	0.423	0.007	0.042	−4.906
(2 0 2)	34.105	0.337	0.006	0.045	−5.133
(3 1 0)	39.798	0.412	0.007	0.062	−4.932
(2 2 2)	46.755	0.378	0.007	0.086	−5.018
(2 1 3)	49.55	0.365	0.006	0.097	−5.053

It can be noted that, as the concentration of Mn-dopant increases, the intercept systematically changes as −5.8445, −5.8497, −5.8539, −5.8368, −5.8123, and −5.7039. The crystallite sizes were 47.36, 47.59, 47.81, 46.99, 45.84, and 41.14 nm, respectively (Table 2); for M1 and M2, the crystallite size increased, but for M3, M4, and M5, a decrease is observed.

**Table 2:** Average crystallite size (D) of pure and doped Hap using a modified Scherrer equation

Sample	e^(LnKλ/D)^ = 0.89λ/D	D(nm)
M0	e^−5.1315^ = 0.005907	23.67
M1	e^−5.1565^ = 0.005762	23.79
M2	e^−5.1608^ = 0.005737	23.89
M3	e^−5.1438^ = 0.005835	23.49
M4	e^−5.2379^ = 0.005311	24.33
M5	e^−5.1091^ = 0.006041	22.69

**Fig. 4:**
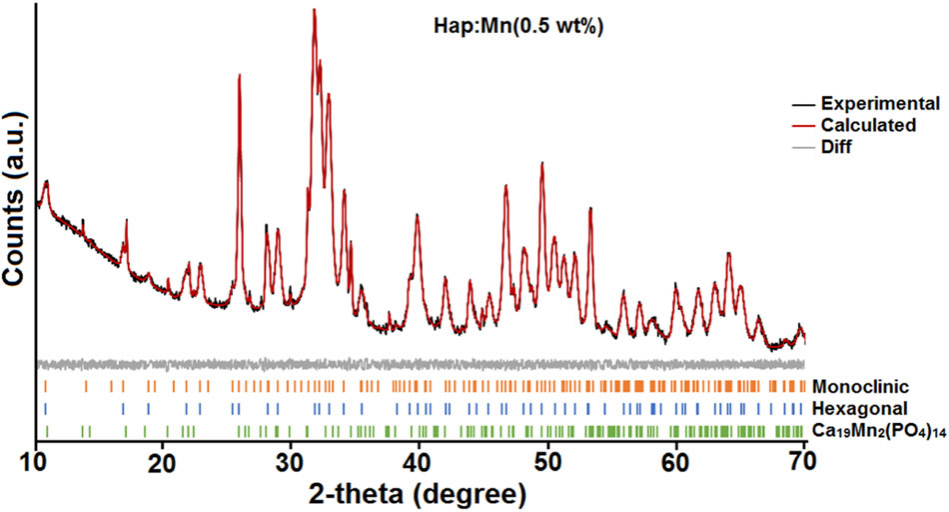
Rietveld analysis from M2 sample

The Rietveld method was used to determine the amount of each crystalline phase present in the samples. This was done by analyzing the data obtained from X-ray diffraction patterns. We specifically analyzed the M2, M3, and M4 samples because the diffraction peaks associated with calcium manganese phosphate were visible. Table 3 displays the wet percentage of each phase. From this result, it can be highlighted that the monoclinic phase has the majority presence, but its wet percent decreased when Mn doped is increased. At the same time, the presence of calcium manganese phosphate is increasing. At the same time, the presence of calcium manganese phosphate is increasing. While the presence of hexagonal Hap presents a slight increase, being minor than the presence of the monoclinic phase. Figure 4 shows the typical Rietveld refinement plot where the goodness of fit is appreciated between the experimental and calculated intensities for the M2 sample [HAp; Mn(0.5 wt%)].

**Table 3:** Percent fraction of each crystalline phase calculated from Rietveld analysis

Sample	Hexagonal Hap (% fraction)	Monoclinic Hap (%fraction)	Ca_19_Mn_2_(PO_4_)_14_ (% fraction)
M2	10.74	81.42	7.84
M3	11.17	75.26	13.57
M4	12.92	64.29	22.79

### Transmission Electron Microscopy (TEM)

**Fig. 5:**
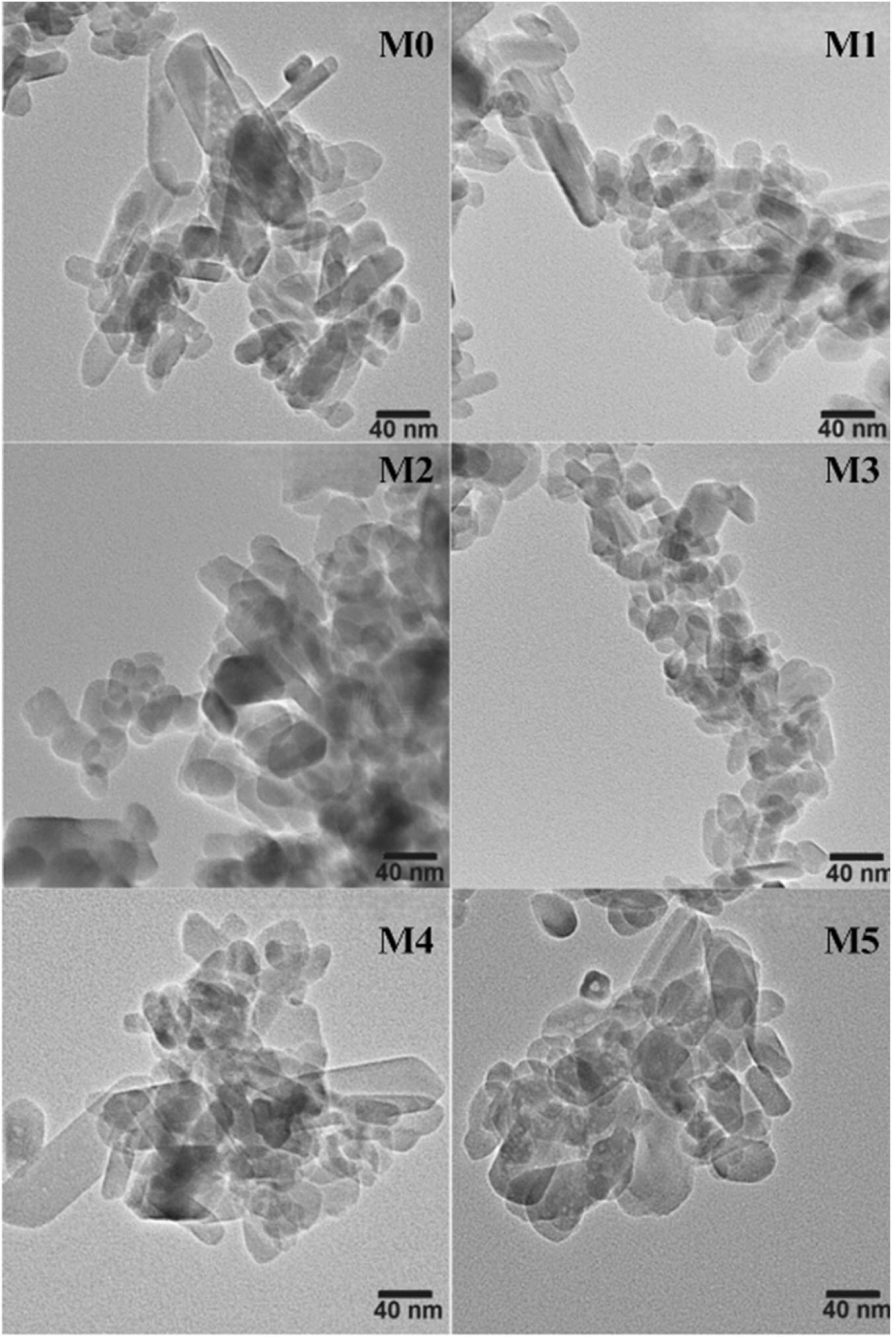
TEM micrographs of pure and Mn doped Hap

Figure 5 shows the TEM micrographs of the Hap samples that are pure and Mn-doped. The pure Hap (M0) shows the formation of rod-shaped structures that range from 5 to 45 nm in diameter and 10 to 90 nm in length. M1 sample also shows rods shape structure with a diameter from 8 to 35 nm and length between 10 and 95 nm. In the M2 sample, rods with diameters between 5 and 50 nm and from 15 to 90 nm in length were observed. The same figure shows that the TEM micrographs for the other doped Hap samples (M3, M4, and M5) showed the presence of nanorod structures with different sizes. Noticeably, in all samples, there are two distributions of particle sizes; one corresponds to nanorods with several tens of nanometers in length, and the other in which the diameter and length are very close. However, the abundance of each one depends on the concentration of Mn-dopant in Hap.

Energy-dispersive X-ray spectroscope (EDS) analysis was used for the pure and Mn-doped Hap to determine the chemical elemental composition (atomic %) and the Ca/P ratio, as is shown in Table 4. The synthesized samples showed a higher ratio of Ca/P concerning the stoichiometric Hap (Ca/P ratio = 1.67), indicating that these samples are rich in calcium. It is also possible to observe a systematic increase of Mn-dopant. Although the ratio Ca/P was greater concerning the stoichiometric, these doped Hap materials could be considered bioabsorbable, considering that bioceramics are considered insoluble for a ratio of 2.0.

**Table 4:** Elemental chemical analysis (atomic %) by EDS of pure and Mn doped samples

Sample	O	p	Ca	Mn	Ca/P
M0	64.59	12.71	22.68	–	1.78
M1	63.86	12.89	23.24	–	1.80
M2	62.20	13.27	24.41	0.10	1.84
M3	59.82	16.45	28.99	0.13	1.76
M4	64.47	12.51	22.74	0.26	1.82
M5	63.55	12.95	23.05	0.44	1.78

### Characterization by Fourier Transform Infrared Spectroscopy (FTIR)

**Fig. 6:**
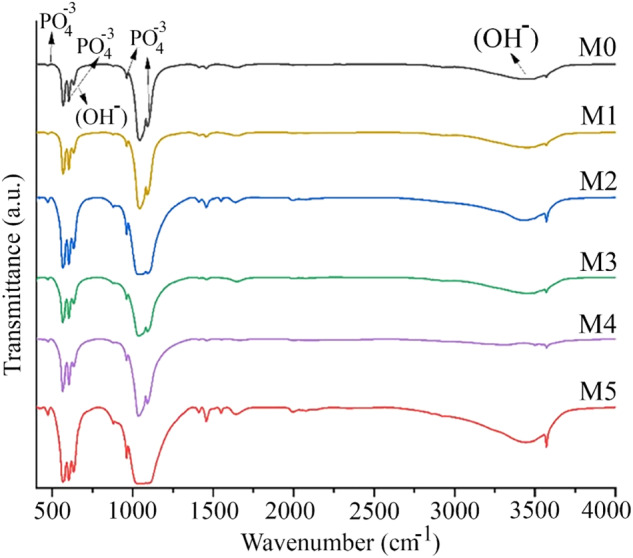
Infrared spectrum (FTIR) of Hap samples doped with MnCl_2_

The FTIR analysis allowed identifying the functional groups of the pure and Mn-doped samples, as illustrated in Fig. 6. FTIR spectra show bands at 636 and 3572 cm^−1^, confirming the stretching vibrations of the OH- group characteristic of the Hap. Likewise, the positions of the bands from 964 to 1200 cm^−1^ correspond to a symmetrical vibration of phosphate bending (O-P-O). In addition, bands with lower intensity observed at 3681, 3655, and 3628 cm^−1^ corresponding to the mode of surface stretching O-H of the P-OH groups, which are formed by protonation of the PO_4_^−3^ions of the surface to compensate for the deficiency of cations of Ca^+2^ in the Hap. Regarding this band, it is possible to observe that the intensity at 3572 cm^−1^ decreases when the dopant concentration increases. Likewise, the bands at 3628 and 3655 cm^−1^ weaken. In that regard, Joris et al. reported that the P-OH groups of the acid surface exhibit a displacement or elimination and appear at a higher wavenumber, such as 3681 cm^−1^ [60]. Therefore, the disappearance of the P-OH bands from the surface suggests the incorporation of Mn^+2^ ions and can increase the acidity of the P-OH groups of the surface, becoming H^+^ ions and promoting the disappearance of OH- ions. Also, the P-OH groups can be constituted by acid ions on the surface [56, 61]. Thus it is, they are converted from PO_4_^−3^ ions to maintain the general charge balance of calcium-deficient; this suggests not only the exchange of calcium ions characteristic of Hap, as is well known, but also the protons of P-OH groups for Mn^+2^ ions.

**Fig. 7:**
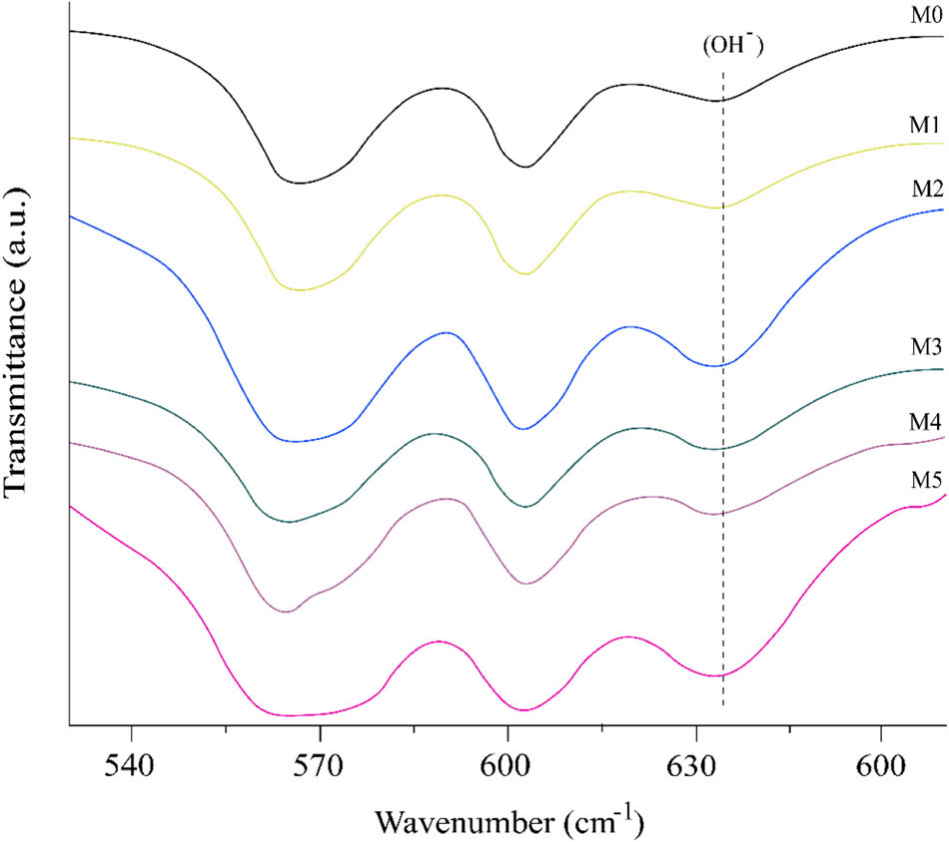
Infrared spectrum (FTIR) of Hap samples doped with MnCl_2_, in a range from 535 to 665 cm^−1^

Venkateswarlu et al. suggested that the absorption bands at 632 and 3571 cm^−1^ for vibrational and stretching modes of hydroxyl vibrations joined to the band at 962 cm^−1^ for phosphate can be used to indicate the crystallinity of Hap (Venkateswarlu et al., 2010). It is interesting to note that the FTIR results show a slight band decrease when the Mn^-^dopant increases (Figs. 6 and 7); this indicates a decrease in the crystallinity of doped Hap, suggesting that Mn^+2^ ions are being incorporated into the crystalline array of the Hap.

### Cellular viability

**Fig. 8:**
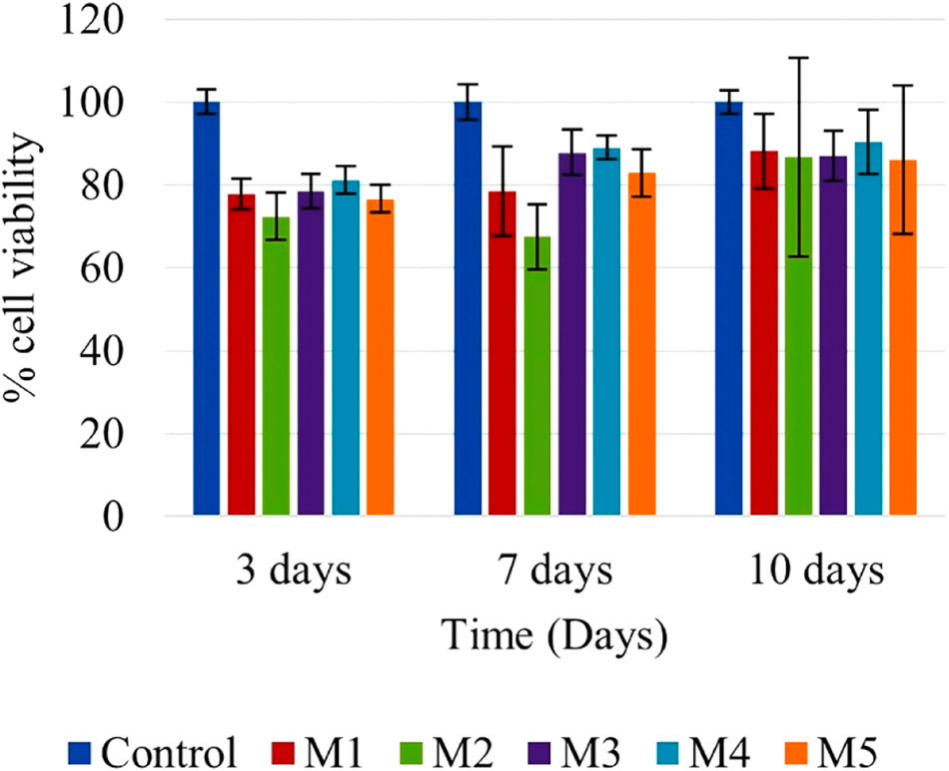
Graph determined by Alamar Blue trial after 3, 7 and 10 days

In vitro, cell viability of HDPSCs cultured at 3, 7, and 10 days was quantitatively evaluated using the Alamar Blue assay. The graph of Hap doped with Mn (Fig. 8) shows that the experimental groups mostly have a percentage higher than 75% cell viability. For the Hap:0.1%Mn sample (M1), a viability of 81.49% ± 5 concerning pure Hap (Control) is obtained; in the case of the Hap:0.5%Mn and Hap:1%Mn samples (M2 and M3, respectively), a viability of 75.55% ± 10 and 84.47% ± 5 is observed, respectively. In the Hap:1.5%Mn sample (M4), the viability of 86.88% ± 5 is appreciated. Finally, in the case of Hap:2%Mn (M5), the viability of 81.92% ± 4 is exposed. In contrast, ISO 10993-5 states that more than 30% of the decrease in cell viability is cytotoxic. This result shows that a lower presence of Mn-dopant slightly reduced the cellular viability; however, the cell viability could be enhanced with a greater presence of Mn-dopant. Although Mn adding in the synthesis of Hap promotes the growth of calcium manganese phosphate, it impossible to ensure this crystalline phase promotes viability; we believe additional studies are necessary.

### Osteogenic differentiation

**Fig. 9:**
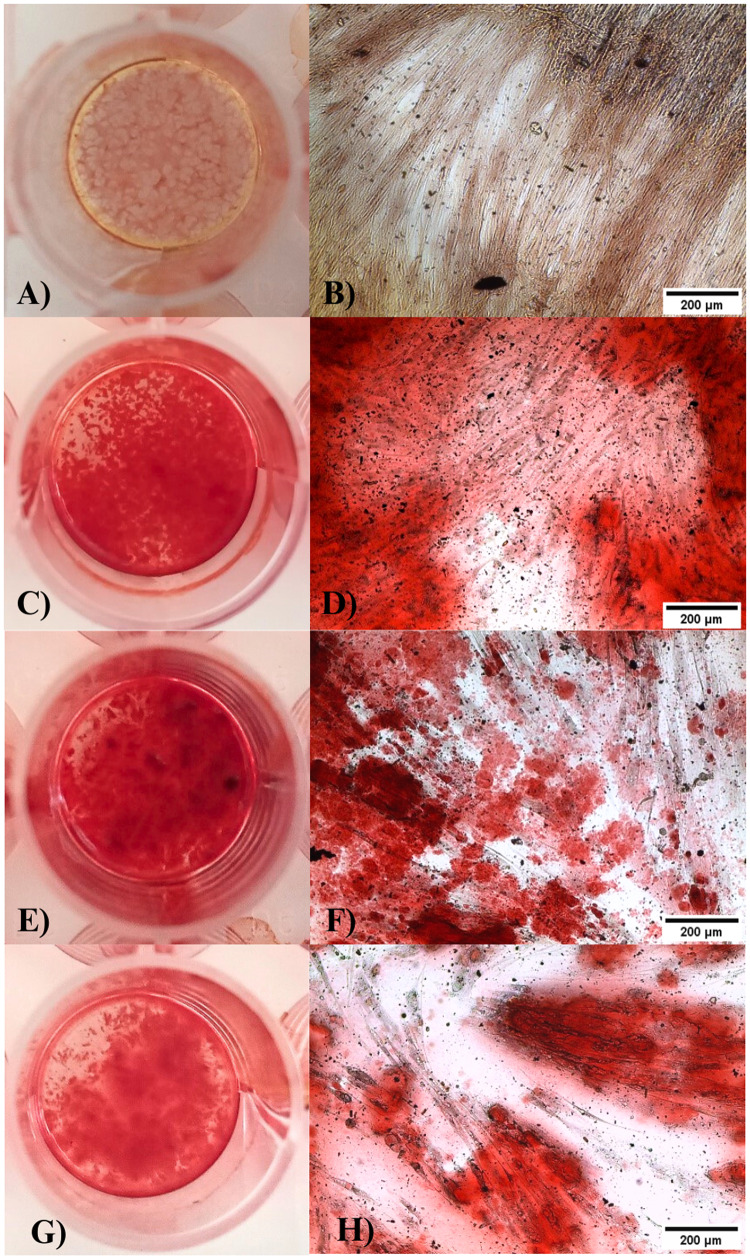
Show Alizarin red stain, DPSCs control group negative **A** macroscopically and **B** microscopically 10×. They show a slightly red mark. DPSCs in the positive control groups **C** and **D** show an intense red mark of calcium deposits. Cultured DPSCs with 1.5% **E**, **F** and 2% **G**, **H** Hap: Mn showed marked deep red by alizarin red staining

Osteogenic differentiation was confirmed by staining calcium deposits using alizarin red. The DPSCs of the negative control group cultured with DMEM showed slightly red staining (Fig. 9A, B). The DPSCs of the positive control group cultured with osteogenic medium showed dense calcium deposits stained intense red by alizarin red staining (Fig. 9C, D), confirming that these cells had undergone osteogenic differentiation. The DPSCs of the experimental groups cultured in Hap doped with Mn (Fig. 9E–H) presented a marked intense red with staining, thus confirming that these cells underwent osteogenic differentiation due to the presence of Hap.

### Immunofluorescence

**Fig. 10:**
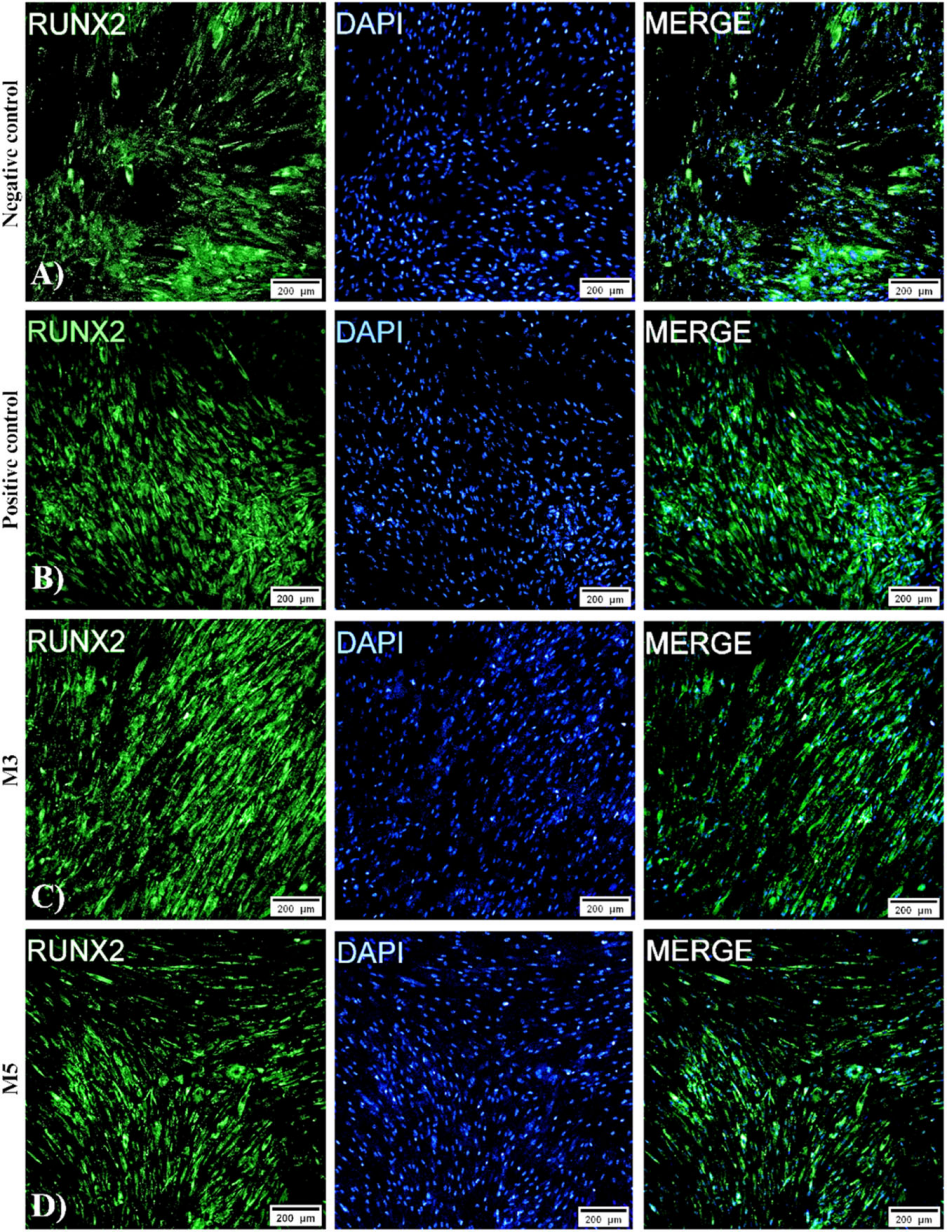
The expression of RUNX2, a key transcription factor associated with osteoblast differentiation, is shown. The immunolabeling was with the primary antibody RUNX2 and the secondary antibody labeled with FITC (green), cell nuclei (blue). Confocal microscopy, 10X lens, 200 μm scale. **A** Negative control, **B** positive control, **C** 1% Hap:Mn and **D** 2% Hap:Mn

**Fig. 11:**
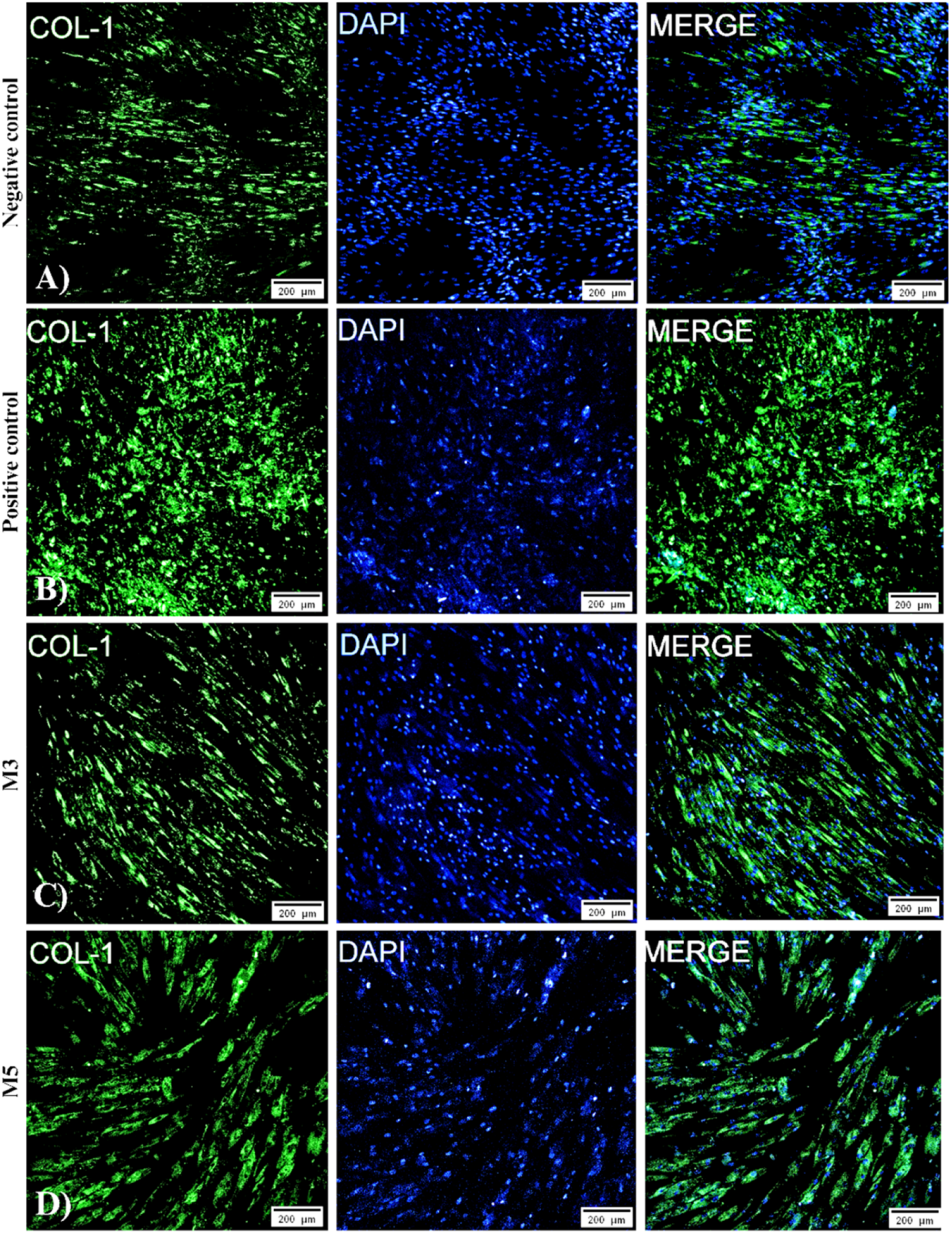
The expression of type I collagen in differentiation to the manganese-doped mediated osteogenic lineage. Primary antibodies of type 1 collagen and Primary. **A** Negative control, **B** positive control, **C** 1% Hap:Mn, and **D** 2% Hap:Mn

RUNX2 immunofluorescence, an early osteogenic differentiation marker, was visualized in all groups. As shown in Fig. 10, the negative control group (Fig. 10A) and Fig. 10B show the positive control group. The M3 sample (Fig. 10C) and M5 (Fig. 10D) were expressed in both groups but at a lower intensity than the positive control group. The cells are considered to be widely dispersed, with polygonal and fibroblastoid morphologies. Type I collagen immunofluorescence, a late osteogenic differentiation marker, was visualized in all groups. As shown in Fig. 11, the negative control group (Fig. 11A) and the positive control group (Fig. 11B) are shown. M3 (Fig. 11C) and M5 (Fig. 11D) were expressed in both groups but at a lower intensity than the positive control group. The cells are considered to be widely dispersed, with polygonal and fibroblastoid morphologies.

## Discussion

The use of dopant materials in the structure of the Hap is of great interest due to the properties that each dopant confers on the material, expanding the range of applications of the Hap. Consequently, by incorporating atoms or molecules of metals, they can increase or improve their application and biomedical properties. In particular, using Mn as a dopant to modify hydroxyapatite’s morphological, crystallographical, and chemical characteristics was carried out several years ago. Table 5 presents a brief review of recent scientific works which present different studies on the influence of the final characteristics of Hap induced by Mn as a dopant.

**Table 5:** Comparison of different investigations associated with Hap-Mn doping

Method	Precursors	Reaction conditions	Mn concentrations	Morphology	Phase
Microwave [61]	Ca (NO_3_)_2_, (NH_4_)_2_HPO_4_, MnCl_2_	800 W 7 min	0, 2 y 4% wt	Rods 110 ± 15 nm	hexagonal Hap Β-TCP
Chemistry [62]	Hap commercial, MnO_2_	(1.3–2.5 MPa)	0.5 a 1% wt	Agglomerates of nanoparticles	Hexagonal Hap
Microwave [63]	Ca (OH)_2_, (NH_4_)_2_HO_2_			Nanoparticles 160–85 nm	Hexagonal Hap
Sol-gel [64]	Ca (NO_3_)_2_, Mn (NO_3_)_2_, (NH_4_)_2_HPO_4_	100 °C de 3–5 hours El gel dried a 340 °C	0.01, 2, 5 y 15%mol	Agglomerates	Hexagonal Hap Β-TCP
Coprecipitation [54]	Ca_5_ (PO_4_)_3_OH, Ca (NO_3_)_2_, (NH_4_) 2HPO_4_, Mn (NO_3_)_2_	600 W 36 min	1 y 3%M	Semi-spherical 120 nm	Hexagonal Hap
Wet Chemical [55]	Ca (OH)_2_, H_3_PO_4_, MnCl_2_	T environment	0.1, 0.2, 0.5 y 1.0% wt	Rods 40–60 nm	Hexagonal Hap Hap-Mn
Wet Chemical [46]	POCH, Ca (OH)_2_, (CH_3_COO)_2_Mn	T environment	0.1, 0.5, 1.0 y 5.0% wt	-------	Hexagonal Hap Β-TCP, α-TCP, Mn_3_O_4_
Coprecipitation [59]	(NH4)2HPO4, Ca(NO3)2⋅4H2O, Mn(NO3)2	T environment 48 horas	1, 5, 10 y 20% mol	Rods 20–30 nm	Hexagonal Hap MnSO_4_.H_2_O

It can be seen from it that there are several methods of synthesis and chemical precursors to obtain hydroxyapatite. It can also be noted that the experimental conditions and the dopant concentration are also different for each study. As a consequence, hydroxyapatite with specific morphology and crystallographic characteristics was reported in each scientific report. Nanoparticles like rods or semi-spherical shapes were obtained, but all synthesized hydroxyapatite present a hexagonal crystalline phase. In this case, using the hydrothermal method and (NH_4_)_2_HPO_4_ and Ca(OH)_2_ as chemical precursors, it was obtained a Hap with nanorod like structures, with an average crystallite size of 23.67 nm and the presence of monoclinic and hexagonal crystalline phases. It also noted an influence of the Mn on the crystallite size and crystallinity of the Hap. This assertion is supported by the cell viability, which showed very low cytotoxicity for all Mn-doped Hap samples. In addition, the staining of calcium deposits using alizarin red showed a positive osteogenic differentiation for the samples, establishing good cell adhesion and biocompatibility.

**Fig. 12:**
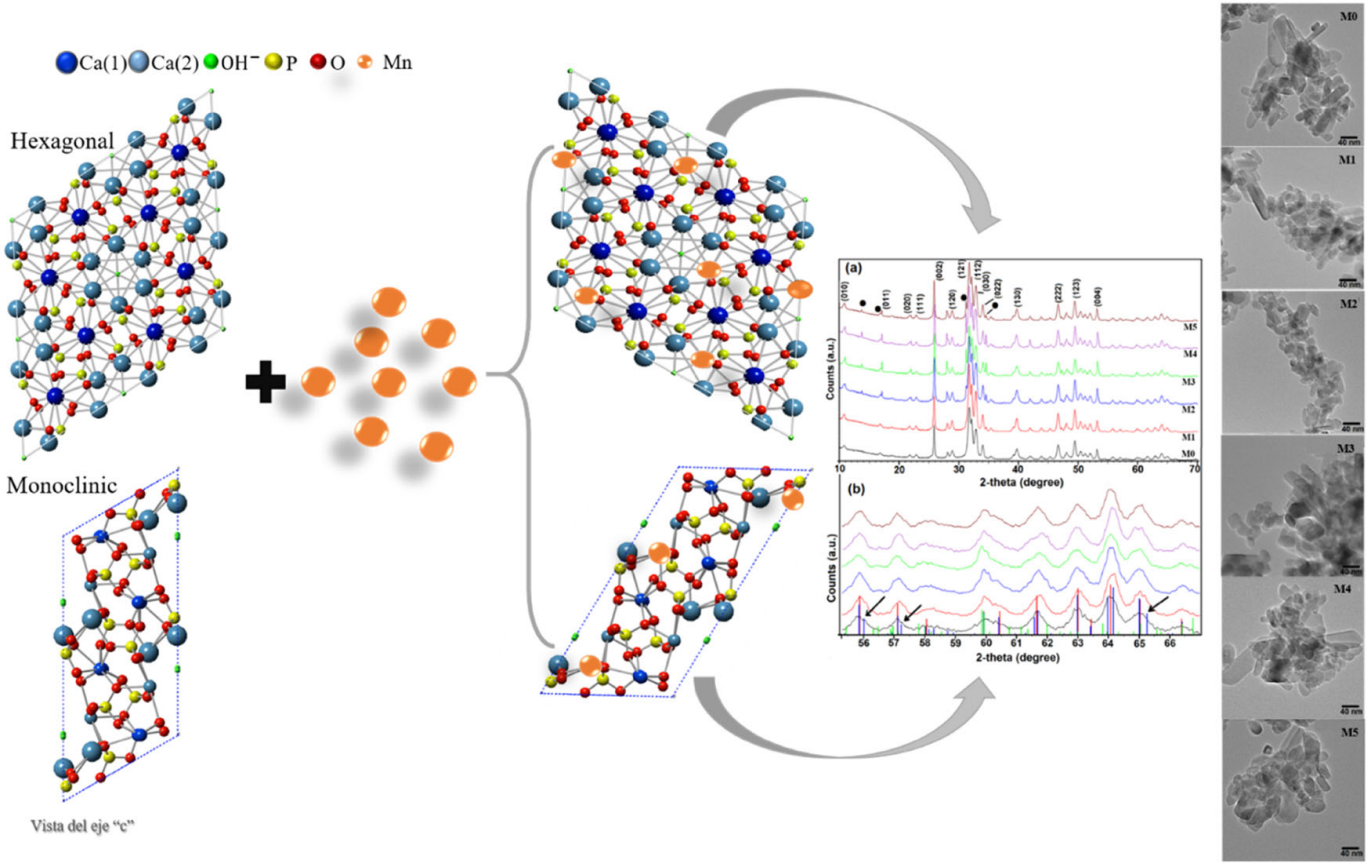
Mechanism of interaction between Mn and Hap, a) M0, b) M1, c) M2, d) M3, e) M4, f) M5

From this, the schematization of the interaction mechanism between Mn and Hap was carried out. Figure 12 shows that the ion exchange occurs with the Ca^+2^ of the Hap, for which there are two sites to affect the ion exchange, which would be with the Ca type 1. The Ca types 2 and 1 are coordinated with nine oxygen atoms. In contrast, Ca type 2 is coordinated with seven oxygen atoms, which favors ion exchange in Ca type 2 since, compared to Ca type 1, less energy would be required to replace it. Similarly, it is essential to note that incorporating Mn does not affect the material’s morphology, maintaining a predominant nanorods morphology.

## Conclusions

The methodology used in the present research for synthesizing Hap favors the formation of nanorods, whose nanosize depends on the Mn concentration. The XRD analysis revealed the presence of hexagonal and monoclinic phases for Hap, with a decrease in the crystallite size due to increased Mn-dopant concentration. This last could be attributed to substituting Mn^+2^ ions with the Ca^+2^ ions of the Hap, modifying the crystal size, which is supported by the model of the mechanism of interaction between Mn and Hap proposed in this work.

In addition, tests conducted on cell viability revealed that the human dental pulp stem cells in the M4 sample had a viability rate of 86.88% ± 5. Suggesting that the doping of Mn to the structure of the Hap allows for improving the biological and structural properties of the Hap; likewise, through the analysis of osteogenic differentiation, it expresses that the incorporation and increase of the concentration of Mn favor the formation of bone cells. These results are of high importance given the wide range of applications of Hap within tissue engineering, demonstrating that the synthesized samples present good cell adhesion, cell viability, biocompatibility, and osteoinduction properties, being a promising material for the repair and reconstruction of bone defects.

## References

[1] López-Cerero L (2008). Infecciones relacionadas con los implantes dentarios. Enferm Infecc Microbiol Clin.

[2] Awasthi S, Pandey SK, Arunan E, Srivastava C (2021). A review on hydroxyapatite coatings for biomedical application: experimental and theoretical perspectives. J Mater Chem B.

[3] Andrés S, Pulido G, González V, Navarro F, López F, Panadero A (2015). Periimplantitis y mucositis periimplantaria. Factores de riesgo, diagnóstico y tratamiento risk factors, diagnosis and treatment of peri-implant disease: a literature review. Av Periodoncia.

[4] Wisanuyotin T, Paholpak P, Sirichativapee W, Kosuwon W (2022). Allograft versus autograft for reconstruction after resection of primary bone tumors: a comparative study of long-term clinical outcomes and risk factors for failure of reconstruction. Sci Rep.

[5] Huang J, Best S. Ceramic biomaterials for tissue engineering. In: Aldo R. Boccaccini, Peter X. Ma and Liliana, editors. Tissue engineering using ceramics and polymers (Third edition). Woodhead Publishing, 2014. p. 3–34.

[6] Baino F, Novajra G, Vitale-Brovarone C (2015). Bioceramics and scaffolds: a winning combination for tissue engineering. Front Bioeng Biotechnol.

[7] Khalaf AT, Wei Y, Wan J, Zhu J, Peng Y, Abdul Kadir SY (2022). Bone tissue engineering through 3D bioprinting of bioceramic scaffolds: a review and update. Life..

[8] Kargozar S, Singh RK, Kim HW, Baino F (2020). “Hard” ceramics for “Soft” tissue engineering: paradox or opportunity?. Acta Biomater.

[9] Du X, Fu S, Zhu Y (2018). 3D printing of ceramic-based scaffolds for bone tissue engineering: an overview. J Mater Chem B..

[10] Chiroff RT, White EW, Weber JN, Roy DM (1975). Tissue ingrowth of replamineform implants. J Biomed Mater Res.

[11] Ruiz-Salgado S, Salado-Leza DE, Reyes-Valderrama MI, Rodríguez-Lugo V (2022). Revisión de compositos de biocerámicas y biopolímeros mediante electrohilado para su uso potencial como andamios para la sustitución de piel. Pädi Boletín Científico de Cienc Básicas e Ingí del ICBI.

[12] Sadat-Shojai Mehdi, Khorasani Mohammad-Taghi, Dinpanah Ehsan (2013). Khoshdargi, Ahmad Jamshidi. Synthesis methods for nanosized hydroxyapatite with diverse structures. Acta Biomater.

[13] Mondal S, Dorozhkin SV, Pal U. Recent progress on fabrication and drug delivery applications of nanostructured hydroxyapatite. Wiley Interdiscip Rev Nanomed Nanobiotechnol. 2018;10:e1504. 10.1002/wnan.1504 29171173

[14] Guo R, Li S, Chen S, Zhang J, Ikoma T, Li X, et al. Synthesis of hydroxyapatite whisker membranes for use as biocompatible and recyclable filters for bacterial removal. J Phys Chem Solids. 2022;170:110901.

[15] Robles-Águila MJ, Reyes-Avendaño JA, Mendoza ME (2017). Structural analysis of metal-doped (Mn, Fe, Co, Ni, Cu, Zn) calcium hydroxyapatite synthetized by a sol-gel microwave-assisted method. Ceram Int.

[16] García-Garduño, Margarita Victoria, Reyes-Gasga J LA (2006). La Hidroxiapatita, Su Importancia En Los Tejidos Mineralizados Y Su Aplicación Biomédica. TIP Rev Especializada en Cienc Químico Biológicas.

[17] Martínez-Valencia AB, Esparza-Ponce HE, Carbajal-De La Torre G, Ortiz-Landeros J (2008). Caracterización estructural y morfológica de hidroxiapatita nanoestructurada: estudio comparativo de diferentes métodos de síntesis. Superficies y Vacío.

[18] Zhong S, Chen J, Li Q, Wang Z, Shi X, Lin K (2017). Assembly synthesis of spherical hydroxyapatite with hierarchical structure. Mater Lett.

[19] An L, Li W, Xu Y, Zeng D, Cheng Y, Wang G (2016). Controlled additive-free hydrothermal synthesis and characterization of uniform hydroxyapatite nanobelts. Ceram Int.

[20] Ribeiro TP, Monteiro FJ, Laranjeira MS (2020). Duality of iron (III) doped nano hydroxyapatite in triple negative breast cancer monitoring and as a drug-free therapeutic agent. Ceram Int.

[21] Mary IR, Sonia S, Viji S, Mangalaraj D, Viswanathan C, Ponpandian N (2016). Novel multiform morphologies of hydroxyapatite: synthesis and growth mechanism. Appl Surf Sci.

[22] Balhuc S, Campian R, Labunet A, Negucioiu M, Buduru S, Kui A (2021). Dental applications of systems based on hydroxyapatite nanoparticles—an evidence-based update. Crystals.

[23] Gshalaev VS, Demirchan AC. Hydroxyapatite: synthesis, properties, and applications. Nova Biomedical; 1st edition; 2012.

[24] Zhu Y, Xu L, Liu C, Zhang C, Wu N (2018). Nucleation and growth of hydroxyapatite nanocrystals by hydrothermal method. AIP Adv.

[25] Ferraz MP, Monteiro FJ, Manuel CM (2018). Hydroxyapatite nanoparticles: a review of preparation methodologies. J Appl Biomater Biomech [Internet].

[26] Ortega J, Villarreal N, Villa H (2004). Visualización cristalográfica de la hidroxiapatita. Ingías.

[27] Rodríguez-Lugo V, Angeles-Chavez C, Hernandez M (2003). Synthesis of hydroxylapatite from sand dollar and β-tricalcium phosphate by solid-state method. Mater Manuf Process.

[28] Fihri A, Len C, Varma RS, Solhy A (2017). Hydroxyapatite: a review of syntheses, structure and applications in heterogeneous catalysis. Coord Chem Rev.

[29] Tayyebi S, Mirjalili F, Samadi H, Nemati A (2015). A review of synthesis and properties of hydroxyapatite/alumina nanocomposite powder. Chemistry Journal.

[30] Rodríguez-Lugo V, Angeles-Chavez C, Mondragon G, Recillas-Gispert S, Castaño VM (2016). Synthesis and structural characterization of hydroxyapatite obtained from CaO and CaHP04 by a hydrothermal method. Mater Res Innov.

[31] Rodríguez-Lugo V, Sanchez Hernández J, Arellano-Jimenez MJ, Hernández-Tejeda PH, Recillas-Gispert S (2005). Characterization of hydroxyapatite by electron microscopy. Microsc Microanalysis.

[32] Rodríguez-Lugo V, Angeles C, De la Isla A, Castano VM (2015). Effect of bio-calcium oxide on the morphology of hydroxyapatite. Int J Basic Appl Sci.

[33] Rodríguez-Lugo V, Salinas-Rodríguez E, Vázquez RA, Alemán K, Rivera AL. Hydroxyapatite synthesis from a starfish and β-tricalcium phosphate using a hydrothermal method. RSC Adv. 2017;7:7631–9. http://xlink.rsc.org/?DOI=C6RA26907A.

[34] Rodríguez-Lugo V, Karthik TVK, Mendoza-Anaya D, Rubio-Rosas E, Villaseñor Cerón LS, Reyes-Valderrama MI (2018). Wet chemical synthesis of nanocrystalline hydroxyapatite flakes: effect of pH and sintering temperature on structural and morphological properties. R Soc Open Sci.

[35] Sánchez-Campos D, Mendoza-Anaya D, Reyes-Valderrama MI, Esteban-Gómez S, Rodríguez-Lugo V (2020). Cationic surfactant at high pH in microwave HAp synthesis. Mater Lett.

[36] López-Ortiz S, Mendoza-Anaya D, Sánchez-Campos D, Fernandez-García ME, Salinas-Rodríguez E, Reyes-Valderrama MI (2020). The pH effect on the growth of hexagonal and monoclinic hydroxyapatite synthesized by the hydrothermal method. J Nanomater.

[37] Sánchez‐campos D, Valderrama MIR, López‐ortíz S, Salado‐leza D, Fernández‐garcía ME, Mendoza‐anaya D (2021). Modulated monoclinic hydroxyapatite: the effect of ph in the microwave assisted method. Minerals..

[38] Ortiz SL, Lugo VR, Salado-Leza D, Reyes-Valderrama MI, Alcántara-Quintana LE, González-Martínez P (2021). Dy2O3-unpurified hydroxyapatite: a promising thermoluminescent sensor and biomimetic nanotherapeutic. Appl Phys A.

[39] Villaseñor Cerón LS, Rodríguez Lugo V, Arenas Alatorre JA, Fernández-Garcia ME, Reyes-Valderrama MI, González-Martínez P (2019). Characterization of hap nanostructures doped with AgNp and the gamma radiation effects. Results Phys.

[40] Bryappa K, Yoshimura M. Hydrothermal synthesis and growth of coordinated complex crystals (part I). In: K Byrappa, Masahiro Yoshimura, editors. Handbook of hydrothermal technology (1st edition). William Andrew Publishing. 2001. p. 415–617.

[41] Sadat-Shojai M, Khorasani M-T, Dinpanah-Khoshdargi E, Jamshidi A. Synthesis methods for nanosized hydroxyapatite with diverse structures. 2013. http://ac.els-cdn.com/S1742706113001840/1-s2.0-S1742706113001840-main.pdf?_tid=be32d994-8e51-11e7-9a25-00000aab0f01&acdnat=1504186920_a67ea1ba82ccda3783aecdf8ddd4b2a5. 10.1016/j.actbio.2013.04.012 23583646

[42] Liu J, Ye X, Wang H, Zhu M, Wang B, Yan H (2003). The influence of pH and temperature on the morphology of hydroxyapatite synthesized by hydrothermal method. Ceram Int.

[43] Brown PW, Constantz B. Hydroxyapatite and related materials (1st edition). CRC Press. 1994.

[44] Yoshimura M, Byrappa K (2008). Hydrothermal processing of materials: past, present and future. J Mater Sci.

[45] Vadalà G, Russo F, Ambrosio L, Denaro V. Bioceramics and biocomposites in spine surgery. In: Antoniac, I., editor. Handbook of bioceramics and biocomposites. Springer, Cham. 2016. pp. 967–987.

[46] Paluszkiewicz C, Ślósarczyk A, Pijocha D, Sitarz M, Bućko M, Zima A (2010). Synthesis, structural properties and thermal stability of Mn-doped hydroxyapatite. J Mol Struct.

[47] Li Y, Nam CT, Ooi CP. Iron(III) and manganese(II) substituted hydroxyapatite nanoparticles: characterization and cytotoxicity analysis. J Phys Conf Ser. 2009;187:012024.

[48] Oriňaková R, Oriňak A, Kupková M, Hrubovčáková M, Markušová-Bučková L, Giretová M (2015). In vitro degradation and cytotoxicity evaluation of iron biomaterials with hydroxyapatite film. Int J Electrochem Sci.

[49] Mendoza-Anaya D, Flores-Díaz E, Mondragón-Galicia G, Fernández-García ME, Salinas-Rodríguez E, Karthik TVK (2018). The role of Eu on the thermoluminescence induced by gamma radiation in nano hydroxyapatite. J Mater Sci Mater Electron.

[50] Villaseñor-Cerón LS, Reyes-Valderrama MI, López-Ortiz S, Salinas- Rodríguez E, Rodriguez-Lugo V (2021). El pH como parámetro en la síntesis de hidroxiapatita y cloroapatita a partir del método hidrotermal asistido por microondas. Pädi Boletín Científico de Cienc Básicas e Ingí del ICBI.

[51] Ortiz SL, Rodríguez-Lugo V, Villaseñor-Cerón LS, Reyes-Valderrama MI, Salado-Leza DE, Mendoza-Anaya D (2020). El Potencial de la Hidroxiapatita Dopada como Sensor Termoluminiscente de Radiación ionizante. Pädi Boletín Científico de Cienc Básicas e Ingí del ICBI.

[52] Rodríguez-Lugo V, Salado-Leza DE, Ortiz SL, Mendoza-Anaya D, Villaseñor-Cerón LS, Reyes-Valderrama MI (2020). Revisión de la Hidroxiapatita Nanoestructurada como Alternativa para Tratamiento de Cáncer. Pädi Boletín Científico de Cienc Básicas e Ingí del ICBI.

[53] Sopyan I, Ramesh S, Nawawi NA, Tampieri A, Sprio S (2011). Effects of manganese doping on properties of sol-gel derived biphasic calcium phosphate ceramics. Ceram Int.

[54] Medvecký Ľ, Štulajterová R, Parilák Ľ, Trpčevská J, Ďurišin J, Barinov SM (2006). Influence of manganese on stability and particle growth of hydroxyapatite in simulated body fluid. Colloids Surf A Physicochem Eng Asp.

[55] Pandya HM, Anitha P (2015). Influence of manganese on the synthesis of nano hydroxyapatite by wet chemical method for in vitro applications. Am J Phytomed Clin Therapeutics.

[56] Kandori K, Yamaguchi Y (2017). Synthesis and characterization of Mn- doped calcium hydroxyapatite particles. Phosphorus Res Bull.

[57] Nawawi NA, Sopyan I, Ramesh S, Afzeri. Phase behaviour of manganese-doped biphasic calcium phosphate ceramics synthesized via sol-gel method. Asia-Pacific J Chem Eng. 2011;6:823–31.

[58] Mayer I, Cuisinier FJG, Gdalya S, Popov I (2008). TEM study of the morphology of Mn2+ -doped calcium hydroxyapatite and β-tricalcium phosphate. J Inorg Biochem.

[59] Liu H, Cui X, Lu X, Liu X, Zhang L, Chan TS (2021). Mechanism of Mn incorporation into hydroxyapatite: Insights from SR-XRD, Raman, XAS, and DFT calculation. Chem Geol.

[60] Joris SJ, Amberg CH (1971). Nature of deficiency in nonstoichiometric hydroxyapatites. I. Catalytic activity of calcium and strontium hydroxyapatites. J Phys Chem.

[61] Anwar A, Akbar S (2018). Novel continuous microwave assisted flow synthesis of nanosized manganese substituted hydroxyapatite. Ceram Int.

[62] Ramesh S, Tan CY, Peralta CL, Teng WD (2007). The effect of manganese oxide on the sinterability of hydroxyapatite. Sci Technol Adv Mater.

[63] Kanchana P, Sudhan N, Sekar C, Neri G (2018). Manganese doped hydroxyapatite nanoparticles based enzyme-less electrochemical sensor for detecting hydroquinone. J Nanosci Nanotechnol.

[64] Nawawi NA, Sopyan I, Ramesh S, Afzeri. Phase behaviour of manganese-doped biphasic calcium phosphate ceramics synthesized via sol-gel method. Asia Pacific J Chem Eng. 2011;6:823–31.

